# Nitrofurantoin for the treatment of uncomplicated urinary tract infection in female patients: the impact of dosing regimen, age, and renal function on drug exposure

**DOI:** 10.1007/s00228-023-03507-2

**Published:** 2023-06-02

**Authors:** A. A. van Driel, A. E. Muller, R. A. Wijma, E. E. Stobberingh, A. Verbon, B. C. P. Koch

**Affiliations:** 1 Department of Medical Microbiology and Infectious Diseases, Erasmus University Medical Centre, Dr. Molewaterplein 40, 3015 Rotterdam, The Netherlands; 2 Department of Medical Microbiology, Haaglanden Medisch Centrum, The Hague, The Netherlands; 3 Department Infectious Disease Control, Municipal Public Health Service Rotterdam-Rijnmond (GGD Rotterdam), Rotterdam, The Netherlands; 4 Department of Hospital Pharmacy, Erasmus University Medical Centre, Rotterdam, The Netherlands; 5 Center for Antimicrobial Treatment Optimization Rotterdam (CATOR), Rotterdam, The Netherlands

**Keywords:** Nitrofurantoin, Uncomplicated urinary tract infection, Drugs exposure, Antibiotic dose regimen, Age and renal function

## Abstract

**Purpose:**

The aim of this study is to determine nitrofurantoin exposure in female patients with different age and renal function with complaints of an uncomplicated UTI. Also the nitrofurantoin exposure in relation to the dosage regimen will be studied.

**Methods:**

Eight general practitioners (GP) participated in the study and included 38 patients with symptoms of an uncomplicated UTI, treated either with a dose of 50 mg q6h or 100 mg q12h, upon the discretion of the GP. Nitrofurantoin exposure was quantified in the patient’s 24-h urine samples by UHPLC-UV and the area under the curve was calculated.

**Results:**

The 38 patients provided a range of 2–17 urine samples. The urine nitrofurantoin exposure was 1028 mg h/L for the patients receiving 50 mg q6h and 1036 mg h/L for those treated with 100 mg q12h (*p* = 0.97) and was not affected by age and eGFR (*p* = 0.64 and *p* = 0.34, respectively).

**Conclusion:**

The data obtained do not support the discouragement of nitrofurantoin use in the elderly and in patients with impaired renal function. Since only a small number of patients were included, a larger study with more patients is warranted to evaluate nitrofurantoin exposure and adverse effects.

## Introduction

An uncomplicated urinary tract infection (UTI) is one of the most frequently recorded diagnoses in female general practice patients. In more than 70% of the cases, the causative uropathogen is *Escherichia coli* (*E. coli*) [1, 2]. The worldwide increase in antibiotic resistance affects optimal (oral) treatment of this common infection [3, 4]. Therefore, there is growing interest to use older agents such as nitrofurantoin. This compound has been successfully used over more than 60 years for the treatment of uncomplicated UTI in women without notable increase in antibiotic resistance [5–7]. Nitrofurantoin is the first choice antibiotic for uncomplicated UTI in several countries such as Germany, The Netherlands, the USA, and Australia [8–12]. However, use in the elderly (age > 65 years), in patients with impaired renal function, and in women beyond 38 weeks of pregnancy and/or in the first week of breast feeding is discouraged [6].

The compound is available in macrocrystalline form and as slow release formulation in different countries. Commonly used dose regimens for uncomplicated UTI are Furadantin^®^/Macrodantin^®^ 50 mg q6 h and Furabid^®^/Macrobid^®^ 100 mg q12 h (slow release) [13–16]. The twice daily administration for the long-acting formulation is facilitating patient compliance, but it is unknown whether one of these regimens is preferable over the other in terms of antibiotic exposure. Nitrofurantoin has a broad antibacterial spectrum including *Enterococcus faecalis* and extended spectrum beta-lactamase producing *E. coli.* There is no cross resistance with other antibiotics and the compound reaches only a high concentration in the urine (after 4 h at pH < 8), resulting in minimal side effects [17–19].

Nitrofurantoin was introduced before the European Medicine Agency (EMA) required for the registration pharmacokinetics (PK) and pharmacodynamics (PD) data to show the safety and effectiveness of the compound against certain pathogens to treat specific diseases [5]. As a consequence, PK and PD data are scarce [20, 21].

Newer guidelines [8–12] decreased the eGFR limit below which nitrofurantoin should not be prescribed, from 50 to 30 mL/min based on the results of Bains et al. showing similar effectiveness under 50 mL/min and side effects as with 30 mL/min [22].

In this study, we describe the urinary exposure to nitrofurantoin and the clinical outcome for two commonly used dose regimens, 50 mg q6 h and 100 mg q12 h in relation to age and renal function in females with an uncomplicated UTI visiting the GP. Also, the possible different exposure between the two dose regimes will be presented.

## Methods

### Study design and patient population

Patients were recruited at 8 general practitioners (GP) in the Rotterdam area. The study was approved by the ethics committee of the Erasmus Medical Center (MEC-2017–526). Patients, who visited the GP and needed treatment with nitrofurantoin for a suspected or proven uncomplicated UTI according to standard clinical care, were asked to participate in the study. Upon agreement, a written informed consent was obtained from the participants. The treatment was in line with the national guidelines for GPs for uncomplicated UTI, i.e., 50 mg (Furadantin^®^/Macrodantin^®^) q6 h or 100 mg (Furabid^®^/Macrobid^®^) q12 h during 5 days orally [10]. Inclusion criteria were women of ≥ 18 years, with a known estimated creatinine clearance or if not available, permission from the patient to determine the creatinine clearance during the study. Exclusion criteria were male patients prescription of any antibiotic 1 week before study inclusion, pregnancy of > 38 weeks, first week of breast feeding, patients having porphyria or an allergy for nitrofurantoin, and patients with an estimated creatinine clearance of ≤ 30 mL/min. We included only women as in general practice most patients with uncomplicated urinary tract infection are females.

### Sample collection

Patients were requested to collect urine samples for 24 h on one of the following days: 2, 3, 4, or 5 of treatment ensuring that steady-state situation was reached. On these days, patients recorded in a diary the date, time, and volume of each void. Five milliliters of each void was transferred to a 50-mL container covered with aluminum foil to protect the sample from daylight. Total daily volume of urine was measured by a delivered container. Because of short-term stability at 4 °C, all urine samples were stored in the patients’ freezer (at approximately − 20 °C) for a maximum of 48 h to guarantee stability. At the end of the treatment course, the written dairy and all samples were transferred in a cooler bag to the laboratory and stored at − 80 °C until further analysis.

### Quantification of nitrofurantoin in urine

Nitrofurantoin concentrations were quantified using ultra-high-performance liquid chromatography (UHPLC) with ultra violet (UV) detection at a wave length of 369 nm, according to Food and Drugs Administration (FDA) guidelines [23]. From each collected sample, 100-µL urine was used for sample preparation using liquid–liquid extraction. Linearity was assessed over a concentration range from 4 to 200 mg/L. In case the concentration exceeded the upper level of this concentration range during the initial analysis, samples were diluted with drug-free standard urine. Concentrations below the lower level of this concentration range were recorded as < 4 mg/L (lower limit of quantification). Urine samples were confirmed to be stable for at least 7 days at 4 °C and for 2 years at − 20 °C and at − 80 °C [24].

### Pharmacokinetic and data analysis

The area under the concentration time (AUC) curve in steady state was calculated over 24 h (AUC_0-24h_,) using the linear trapezoidal approach [25].

For the total population and the groups per dosing regimen, the mean, standard deviation (SD), range, and coefficient of variation (CV, %) of the patient demographics and the AUC_0-24h_ were calculated. The AUC_0-24h_ values were compared between the two dosing regimens using an unpaired, two-tailed *t*-test. A *p*-value ≤ 0.05 (two-sided) was considered statistically significant. The following covariates’ contribution to AUC_0-24h_ was investigated: renal function, defined as the determined glomerular filtration rate (eGFR) calculated with the CKD-EPI equation; urinary output, presented as the total volume of urine excreted in 24 h (mL), the number of voids, and the nitrofurantoin excretion rate (mg/h)*.* To study the effect of age, the population was divided for practical reasons into two groups of ≤ 50 years and > 50 years of age. For the renal function, three groups were used: 50–59 mL/min, 60–89 mL/min, and ≥ 90 mL/min (eGFR lower than 50 was not available among the patients, so the lowest category in 50–59 mL/min).

Effectiveness of the treatment was evaluated by asking the GP or nurse whether the patient returned within 2 and 4 weeks after finishing the nitrofurantoin course reporting new or persistent UTI symptoms like dysuria with or without frequency, urgency, suprapubic pain, or hematuria [26]. Also, all patients were called after 2 and 4 weeks to verify the cure of the UTI. In case the patient did not return to their own GP or emergency department, the nitrofurantoin treatment was considered to be effective.

### Safety assessment

Safety evaluation included the collection of adverse events (AEs) and serious AEs (SAEs) reported by the patients to the GP.

## Results

### Study population

In this study, 38 patients participated with a mean age of 57.4 years (range 19–84) and all were Caucasian. The patient demographics are described in Table 1. Seventeen patients had nitrofurantoin 50 mg q6 h and 21 patients 100 mg q12 h. The participants of the last group tended to be younger (53.6 years) than the group of 50 mg q6 regimen (62.1 years), but the difference is not statistically significant (*p* = 0.13). The mean renal function of the two groups was 75.1 mL/min (CV 23.0%, range 50–114) and 79.2 mL/min (CV 21.3%, range 52–125), respectively (*p* = 0.48). The 38 patients collected a total of 336 urine samples. The number of samples received per patient ranged from 2 to 17 (mean 9 samples) and was not related to the dose regimen (*p* = 0.59) (Table 1).

**Table 1:** Patient characteristics

**100 mg q12 h (*n* = 21)**	**Mean**	**SD**	**Range**	**CV**
Age (year)	53.6	17.9	19–80	33.4
Height (cm)	165.5	4.3	151–172	2.6
Weight (kg)	72.0	10.6	56–97	14.7
BMI (kg/m^2^)	26.1	3.7	21–37	14.3
eGFR (mL/min)	79.2	16.8	52–125	21.3
Urine samples (*n*)	8.9	2.8	5–17	31.9

**50 mg q6 h (*n* = 17)**	**Mean**	**SD**	**Range**	**CV**
Age (year)	62.1	14.5	25–84	23.3
Height (cm)	166	6.0	159–182	3.6
Weight (kg)	73.9	13.9	52–102	18.7
BMI (kg/m^2^)	26.8	4.9	19–37	18.3
eGFR (mL/min)	75.1	17.3	50–114	23.0
Urine samples (*n*)	8.4	3.2	2–15	37.8

**Total group (*n* = 38)**	**Mean**	**SD**	**Range**	**CV**
Age (year)	57.4	17.0	19–84	29.6
Height (cm)	165.7	5.1	151–182	3.1
Weight (kg)	72.8	12.2	52–102	16.7
BMI (kg/m^2^)	26.4	4.3	19–37	16.3
eGFR (mL/min)	77.4	17.2	50–125	22.2
Urine samples (*n*)	8.7	3	2–17	34.7

Shows the mean, standard deviation (SD), range, and confidence interval (CV) (%) per dose regimen and total patient group. Patients characteristics are comparable in both groups; age *p* = 0.13, height *p* = 0.76, weight *p* = 0.65, BMI *p* = 0.64, eGFR *p* = 0.48, and *N* urine samples *p* = 0.59

### Pharmacokinetic analysis

The exposure in the urine between the two regimens was not statistically different, with a mean AUC_0-24h_ of 1028 mg h/L in patients taking nitrofurantoin 50 mg q6 h and 1036 mg h/L in those taking the 100 mg q12 h regimen (*p* = 0.97).

**Fig. 1:**
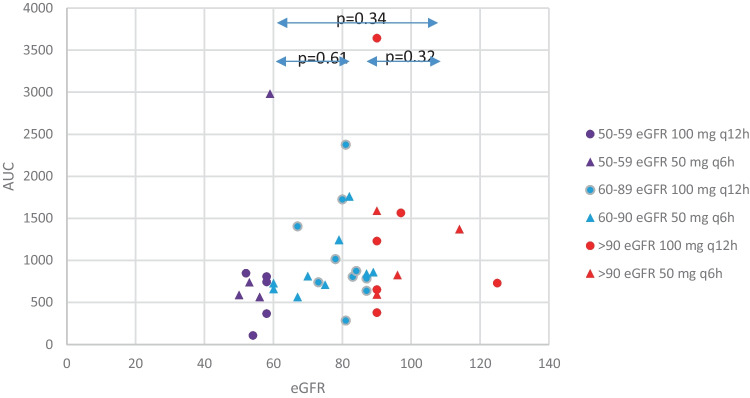
The renal function as presented in three groups versus AUC_0-24h_

The effect of age and renal function (eGFR) on the exposure was studied in the entire study group. The mean values for the AUC_0-24h_ in the 3 eGFR categories (50–59, 60–89, and ≥ 90 mL/min) were 862, 993, and 1260 mg h/L, respectively. The AUC_0-24h_ tended to be higher for a higher eGFR, albeit the differences were not statistically significant (*p* = 0.34) (Fig. 1).

**Fig. 2:**
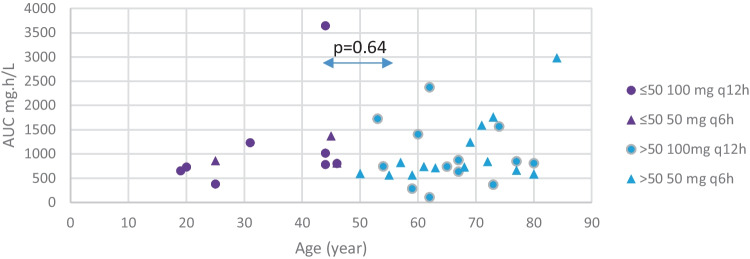
The age versus AUC_0-24h_ for the two age groups

There was also no significant difference in exposure in the urine between the two age groups. In the group with age ≤ 50 years of age (*n* = 11), the mean AUC_0-24h_ was 1118 mg h/L, and in the older age group (> 50 years (*n* = 27)), this value was 997 mg h/L (*p* = 0.64) (Fig. 2).

### Effectiveness of treatment

Two weeks after finalizing the treatment course, 4 patients (11%) went back to their GP with recurrent urinary tract symptoms, two had used the 50 mg dose and two the 100 mg dose. After 4 weeks, an additional patient treated with the 100 mg dose regimen returned to the GP with UTI symptoms. All returned patients were over 50 years, three of them had an eGFR below 60 mL/min. No adverse events were reported by patients to their GP (Table 2). There was no relation between exposure and the patients with treatment failure (*p* = 0.19).

**Table 2:** Patients’ characteristics with recurrent UTI symptoms after 2 or 4 weeks of nitrofurantoin use

**Patient number**	**Age (year)**	**eGFR (mL/min)**	**Dose regimen**	**AUC**_**0-24h**_ **(mg h/L)**	**Time after first dose nitrofurantoin (week)**
1	84	59	50 mg q6h	2985	4
2	61	53	50 mg q6h	740	2
3	67	92	100 mg q12h	639	2
4	73	58	100 mg q12h	368	2
5	62	81	100 mg q12h	2378	2

Within the study, there were no adverse or severe adverse events when using nitrofurantoin.

## Discussion

In this study, the urinary exposure to nitrofurantoin was investigated using the two common dose regimens in women over 18 years of age with complaints of an uncomplicated UTI. There was no significant difference in exposure in the urine between the two regimens. The difference in exposure was not statistically significant between these regimens for age, renal function, and clinical outcome. The outcome was not related to the urine nitrofurantoin exposure nor to the regimen, age, or renal function.

There are not enough consistent data in the literature to establish a PK/PD index for nitrofurantoin. For *Enterobacter cloacae*, a concentration-dependent bactericidal effect was demonstrated, while for *E. coli* and *Klebsiella pneumoniae*, the effect was time dependent [27]. Since the PK/PD index and the minimally required exposure are unknown, the clinical EUCAST (European Committee on Antimicrobial Susceptibility Testing) breakpoint is based on the ECOFF (epidemiological cutoff value) and used in uncomplicated UTI due to *E. coli* only [28]. Here, we could demonstrate no difference in urine exposure to nitrofurantoin in terms of AUC for the two most commonly used dosages. In our study, the PK of nitrofurantoin was independent of the dose and formulation of the dose regimens. These results suggest that the urinary PK of nitrofurantoin is not dependent on dose or formulation alone as was described by ten Doesschate [29]. Possible other contributing factors of PK could be lifestyle, fluid intake, nutrition, and voiding pattern.

We found a high variability in AUC_0-24h_ values in our patients, but it was independent of the formulation and dose. A high variability of PK values was described in several studies in the literature [28–31]. The variation could be explained by differences of included participants (patients or healthy volunteers), dose regimens, or formulations and by differences in renal function, urine frequency, and drugs adsorption. Variation in PK is a common phenomenon for drugs used in the treatment of UTI and not dependent on dose and formulation alone in healthy volunteer studies with no defined time for urine sampling [30–32]. Another possible explanation for the high variability in PK studies reporting urinary exposure is the difference in fluid intake between patients. We did not record the fluid intake to mimic real-life circumstances. The results are in line with the study of Huttner et al. [33]; we did not find differences in urinary exposure between the 50 mg and 100 mg doses, although the authors found a twice as high plasma concentrations with the dose regimen of 50 mg compared with 100 mg. The great variability in number of samples received might explain in part the variation in AUC values in different studies.

In clinical studies, some authors [34] have suggested that the slow dose release (100 mg q12) has higher cure rates than the immediate release capsules, but this is not supported by our data concerning the exposure to nitrofurantoin in the urine.

Two to four weeks after finalizing the course, five patients (13%) went back to the GP as they had persisting or recurrent UTI complaints. This cure rate of 87% was in line with the 70% found in a randomized clinical trial using 100 mg q8 as dose regimen in a comparable patient population [35]. The variation in cure rate might be due to the large individual variability in urinary concentrations resulting in sub-therapeutic concentrations in some patients or the limited number of patients included in the study. The 87% cure rate in our study was based on the number of patients (*n* = 5) returned to the GP with UTI complaints and of the results of the telephone interviews of all patients. No additional treatment failures were mentioned by telephone. Therefore, we considered the 87% cure rate as correct, since patients have their own GP and close communication of emergency rooms to their own GP; no anonymous walk in clinics is present in the Netherlands.

Our study has some strengths and limitations. It is the first study comparing AUC data of two commonly used dose regimens of nitrofurantoin with respect to age, eGFR, urinary exposure, and clinical outcome. As the use of nitrofurantoin was discouraged in the elderly and in patients with impaired renal function, our intended patient population focused on this kind of patients. Since it was an observational study, this limited the inclusion in the study. Other limitations are that we included only female patients as most patients visiting general practitioners with complaints of an uncomplicated urinary tract infection are female patients. Because of common practice, these patients are treated without bacteriological culture of the urine samples. The lack of culture results is another limitation which might influence the cure rate. However, even with these small number of patients, no difference of the urine exposure to nitrofurantoin was found. Furthermore, at the beginning of the study, we wanted to include patients with a wide range of eGFR (starting from 30 mL/min). As the study had a naturalistic design, performed at GPs, we were only able to include women with eGFRs between 50–59, 60–89, and ≥ 90 mL/min, as the GPs were reluctant to prescribe nitrofurantoin to women with an eGFR < 50 mL/min. Therefore, no conclusion can be drawn on the effect of a reduced eGFR (below 50 mL/min) on the urinary exposure of nitrofurantoin.

In conclusion, based on the data of our study, we found that a dose of 50 mg q6h or 100 mg q12 nitrofurantoin was comparable and that there is no preference of one above the other dose regimes. To answer the question as to the applicability of nitrofurantoin in the elderly and in those with impaired renal function, studies with more patients are needed.

## Data Availability

Data is available upon request.

## Declarations

### Transparency declaration

None to declare.
